# The novel use of urinary androgens to optimise detection of the fertile window in giant pandas

**DOI:** 10.1530/RAF-22-0031

**Published:** 2022-06-30

**Authors:** Kirsten S Wilson, Desheng Li, Iain Valentine, Alan McNeilly, Simon Girling, Rengui Li, Yingmin Zhou, Lynn Vanhaecke, W Colin Duncan, Jella Wauters

**Affiliations:** 1 MRC Centre for Reproductive Health, Queen’s Medical Research Institute, University of Edinburgh, Edinburgh, UK; 2 Key Laboratory of SFGA on Conservation Biology of Rare Animals in The Giant Panda National Park, China Conservation and Research Centre for the Giant Panda (CCRCGP), DuJiangYan City, Sichuan Province, China; 3 Zoocraft Ltd., Scotland, UK; 4 RZSS Edinburgh Zoo, Edinburgh, UK; 5 Laboratory of Integrative Metabolomics, Department of Translational Physiology, Infectiology and Public Health, Faculty of Veterinary Medicine, Ghent University, Merelbeke, Belgium; 6 Leibniz Institute for Zoo and Wildlife Research, Department Reproduction Biology, Berlin, Germany

**Keywords:** hormones, non-invasive, estrus, female, breeding, ELISA

## Abstract

**Graphical abstract:**

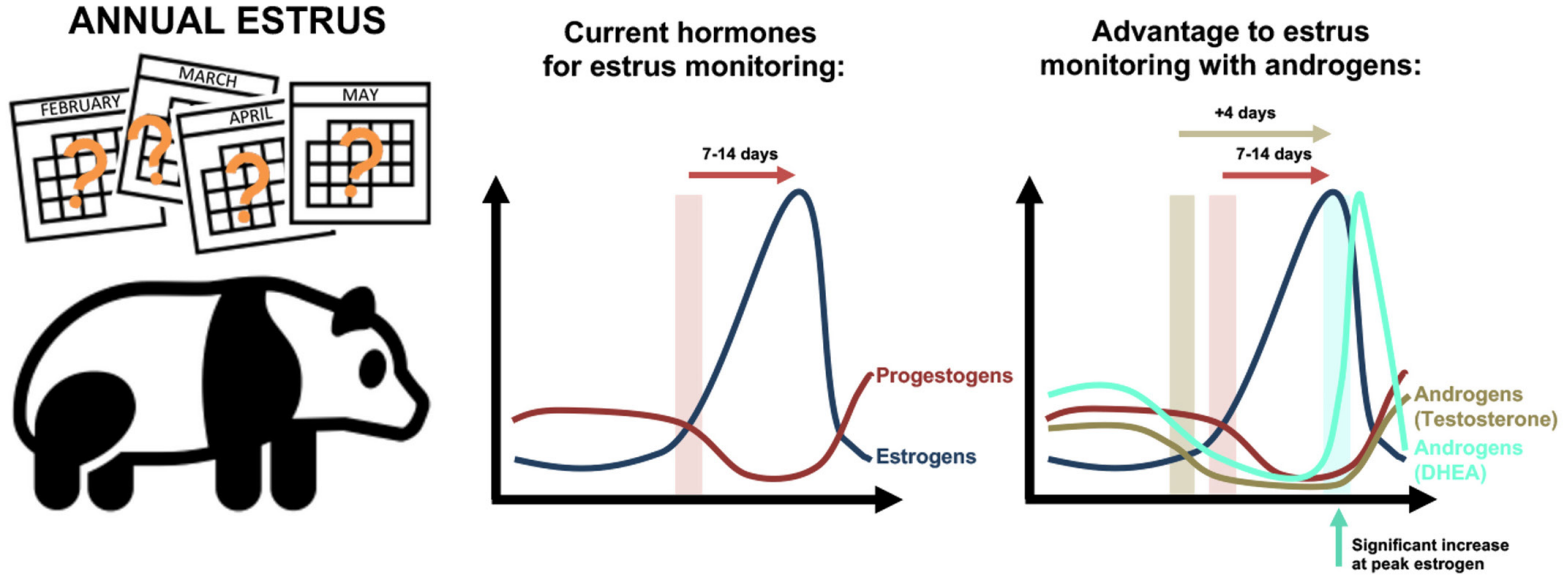

**Abstract:**

Giant pandas are mono-estrus seasonal breeders, with the breeding season typically occurring in the spring. Successful fertilization is followed by an embryonic diapause, of variable length, with birth in the late summer/autumn. There is a need for additional understanding of giant panda reproductive physiology, and the development of enhanced biomarkers for impending proestrus and peak fertility. We aimed to determine the utility of non-invasive androgen measurements in the detection of both proestrus and estrus. Urine from 20 cycles (−40 days to +10 days from peak estrus) from 5 female giant pandas was analyzed for estrogen, progestogens and androgens (via testosterone and DHEA assays), and hormone concentrations were corrected against urinary specific gravity. Across proestrus, estrogens increased while progestogens and androgens decreased – at the point of entry into proestrus, androgens (as detected by the testosterone assay) decreased prior to progestogens and gave 4 days advanced warning of proestrus. At the time of peak estrus, androgens (as detected by the DHEA assay) were significantly increased at the time of the decrease in estrogen metabolites from the peak, acting as an alternative confirmatory indicator of the fertile window. This novel finding allows for enlargement of the preparative window for captive breeding and facilitates panda management within breeding programmes. Androgens allow an enhanced monitoring of giant panda estrus, not only advancing the warning of impending proestrus, but also prospectively identifying peak fertility.

**Lay summary:**

Giant pandas have one chance at pregnancy per year. The 2-day fertile window timing varies by year and panda. This is monitored by measuring the level of estrogens in the urine, which increase, indicating an upcoming fertile period. After 1–2 weeks of increase, estrogens peak and fall, marking the optimal fertile time. We tested other hormones to see if we can predict the fertile window in advance, and the specific fertile time with more accuracy. In 20 breeding seasons from 5 females, we found androgens, usually thought of as male hormones, had an important role. Testosterone gives 4 days advanced warning of estrogens increasing. DHEA identified peak estrogen and the fertile time before needing to see a confirmed decrease in estrogen itself. Therefore, androgens help improve monitoring of the giant panda breeding season, giving early warning of fertility, key in facilitating captive breeding and giant panda conservation.

## Introduction

Giant pandas (*Ailuropoda* *melanoleuca*) are seasonally monoestrus, coming into season during the spring (typically between February and May), and being fertile for only 24–48 h after a 1–2 week period of increasing estrogens (Bonney *et al.* 1982, Lindburg *et al.* 2001, McGeehan *et al.* 2002, Czekala *et al.* 2003, Steinman *et al.* 2006, Kersey *et al.* 2010*a*). Following a short estrus, giant pandas undergo a primary luteal phase, with a small rise in progestogens (assessed by measuring progesterone metabolites) for 60–120 days (Hodges *et al.* 1984, Steinman *et al.* 2006, Zhang *et al.* 2009, Kersey *et al.* 2010*b*), during which an embryonic diapause occurs if fertilization has been successful. A secondary luteal phase follows, which has a more significant progestogen rise and a more consistent duration (45–50 days), which is when fetal development takes place (Steinman *et al.* 2006, Kersey *et al.* 2010*b*). Progestogen measurements cannot be used as a pregnancy marker due to the occurrence of pseudopregnancy in giant pandas; indeed, both the progestogen profile and the behavioural changes are similar in pregnant and non-pregnant females (Monfort *et al.* 1989, Steinman *et al.* 2006, Zhang *et al.* 2009, Kersey *et al.* 2010*a*, 2010*b*, Willis *et al.* 2011, Roberts *et al.* 2018, Wilson *et al.* 2019).

With more giant panda pairs being loaned outside of China as part of international breeding programmes, there is an increased interest in enhancing the understanding of the female reproductive physiology. Recent studies have focussed on determining pregnancy status (Willis *et al.* 2011, Cai *et al.* 2017, Roberts *et al.* 2018, Wilson *et al.* 2019). However, there is an additional need for research into other phases of the reproductive cycle, including the prediction of estrus timing.

Determining an upcoming estrus is monitored by both non-invasive endocrinology and behavioural observations, with the aim being to determine optimal timing for a natural mating (NM) or artificial insemination (AI) (Bonney *et al.* 1982, Monfort *et al.* 1989, Lindburg *et al.* 2001). Endocrinologically, the focus is on measuring estrogens (typically (conjugated) estrone metabolites) in the urine that increase across a 1–2 week period before peak fertility. The duration and magnitude of the increase can vary between females and cycles. In addition, progestogens are measured and concentrations decrease at the time estrogen begin to rise (McGeehan *et al.* 2002, Czekala *et al.* 2003, Kersey *et al.* 2016).

Advance warning of peak fertility is important to allow the appropriate practitioners and equipment for AI to be gathered in a timely manner. At present, the crossover point, where the early rising estrogens surpass the falling progestogen concentrations, is used as a marker of proestrus. Earlier warning of impending proestrus would further help to facilitate logistical planning. Insemination is carried out just after the peak in urinary estrogen concentrations. However, there can be varying estrogen concentrations in sequential samples in individual pandas, and so during real-time monitoring, it can be unclear if a small decrease in estrogens from the previous sample is indicative of the peak having occurred. There is an unmet need for additional non-invasive markers to optimise the timing of insemination.

In other *ursid* species, namely the polar bear (*Ursus maritimus*) and sun bear (*Helarctos malayanus*), androgens have been suggested as better estrus indicators than estrogens (Schwarzenberger *et al.* 2004, Stoops *et al.* 2012). However, polar bears and sun bears show differing reproductive features to giant pandas. Polar bears are induced ovulators, and sun bears are non-seasonal polyestrus breeders. As estrogens are synthesised from androgens, which are stimulated by luteinising hormone (LH), we hypothesised that a decrease in androgens would be a marker of increasing estrogens, and thus impending proestrus, and that increasing androgens would be a steroid marker of the LH surge to help timing of insemination. We aimed to (i) validate assays for urinary testosterone (T) and DHEA in giant panda urine; (ii) determine the utility of androgens in the detection of imminent proestrus; (iii) assess whether androgens can be used to aid the timing of AI during estrus. Herein, we report the assessment of androgen profiles around the fertile window in giant pandas for the first time.

## Methods

### Sample collection

Urine samples are routinely collected from female giant pandas for reproductive monitoring. Samples were aspirated from the floor of the enclosure in the absence of the animal during routine maintenance and were frozen at either −20 or −40 °C. Urine was collected daily until keepers noted proestrus behavioural changes, and then urine was collected multiple times per day when possible. All animal-related work was conducted in line with the relevant national and international guidelines, and no specific ethical approval was required for this study.

Urine samples were collected from five female giant pandas housed at Royal Zoological Society of Scotland (RZSS) Edinburgh Zoo, Scotland (Studbook number (SB) 569), Pairi Daiza, Belgium (SB741), Ouwehands Dierenpark, The Netherlands (SB884), ZooParc de Beauval, France (SB723) and Ähtäri Zoo, Finland (SB941) (Table 1). For this study, the period of interest was defined as 40 days before peak oestrogens (E) until 10 days post peak E. A total of 1350 urine samples were analysed (Table 2) covering 20 cycles (average of 68 samples per cycle), with NM or AI undertaken in 12 of the cycles.

**Table 1:** Summary of the female giant pandas included in this study with location, date of birth, years of cycles, age (in years) at first cycle included in this study, and average body weight at the start of anestrus (the start of the period of interest for this study).

Zoological institution	Panda SB	Date of birth (dd.mm.yy)	Years of cycles	Age (years) at first cycle included	Average weight at start of anestrus (Kg)
RZSS Edinburgh Zoo, UK	SB569	24.08.03	2012, 2013, 2015–2021	8	101.5
Pairi Daiza, Belgium	SB741	07.07.09	2015, 2016, 2018, 2019	5	120.0
Ouwehands Dierenpark, The Netherlands	SB884	05.08.13	2018–2020	4	108.7
ZooParc de Beauval, France	SB723	10.08.08	2017, 2020	8	102.0
Ähtäri Zoo, Finland	SB941	21.09.14	2019, 2020	4	110.3

**Table 2:** Summary of the urine samples available for analysis for each female giant panda – 1350 samples were included with an average of 68 samples per cycle, covering 20 cycles (day −40 to +10 from peak E) from five female giant pandas.

Panda SB	Total number of cycles	Number of samples				
		Anestrus	Proestrus	Estrus	Post-estrus	Total
SB569	9	301	169	42	78	590
SB741	4	73	105	34	64	276
SB884	3	100	55	13	31	199
SB723	2	5	30	6	31	72
SB941	2	71	93	11	38	213
**Total**	**20**	**550**	**452**	**106**	**242**	**1350**

Urine samples from the male giant panda (SB564) housed at RZSS Edinburgh Zoo, Scotland, were also collected and analysed as part of the biological validation of the androgen assays. This panda was discovered to have suspected bilateral testicular tumours in late October 2018, with confirmation and castration performed in early November 2018. A total of 679 urine samples between January 2017 to December 2019 were collected. These represent 445 samples prior to castration and 234 samples following castration.

Samples were transported frozen from their respective zoological parks to the laboratories, and upon the first thaw at the laboratory, the samples were centrifuged (15 min at 2000 ***g*** at room temperature) and assessed for urinary specific gravity (USpG).

### Urinary specific gravity

USpG was used for urinary hormone correction. The USpG of all samples was measured, and the hormones corrected against this as previously described and validated (Wauters *et al.* 2018).

### Measurement of estrogens

Urinary estrogens were assessed using the Arbor Assays DetectX® Enzyme Immunoassay (EIA) Kits for E1G (Estrone-3-Glucuronide; K036-H5; Arbor Assays™, Ann Arbor, Michigan, USA) or E1S (Estrone-3-Sulphate; K031-H5 with the E1S Standard (no. C135), Arbor Assays™, Ann Arbor, Michigan, USA) as previously described (Wauters *et al.* 2018, Wilson *et al.* 2019). Samples were measured at ×10 or ×20 dilution in Arbor Assays assay buffer until proestrus (depending on the USpG value of the sample), and then dilutions between ×100 and ×2000 were measured until after peak E had been reached. Inter- and intra-assay coefficients of variation (CV) were calculated as 12.5 and 4.0%, respectively, for the E1G assay and 10.5 and 6.0% for the E1S assay.

Measurements based on the E1G assay were undertaken on samples analysed by the University of Edinburgh while measurements based on the E1S assay were undertaken on samples analysed by Ghent University. To allow for direct comparisons to be made between the E1G and E1S assay outcomes, E1S assay measures were converted to the E1G assay using a conversion factor (0.381; as described in Wilson *et al.* 2019). For the purpose of this study, results will be presented and converted to E1G-assay measurements.

### Measurement of progestogens

Urinary progestogens were assessed using the Arbor Assays Progesterone DetectX® EIA (K025-H5; Arbor Assays™) as previously described (Wauters *et al.* 2018, Wilson *et al.* 2019). Samples were measured at ×10 dilution in assay buffer. Inter- and intra-assay CV were calculated as 13.8 and 2.0%, respectively.

### Measurement of androgens - testosterone assay

ELISA plates (96-well; Greiner Bio-One, GmbH, Germany) were coated overnight with 7.6 µg/mL donkey anti-rabbit serum IgG (prepared in house from the Scottish Antibody Production Unit, UK) at 4°C in 100 mM sodium bicarbonate buffer (100 µL). Plates were washed twice with wash buffer (300 µL; TBS with 0.05% Tween), then blocked with blocking buffer (220 µL; 0.5% BSA phosphate-buffered saline (PBS)) for 1 h at room temperature. Standards (between 0.03 and 24.3 ng/mL prepared from powdered testosterone (Sigma-Aldrich) in blocking buffer), quality controls (Lyphochek® Fertility Controls, Bio-Rad), and samples, diluted ×5 in assay buffer, were added in duplicate to the plate (16 µL). Testosterone-horse radish peroxidase (84 µL of 1:20,000; no. 12-03 Astra Biotech, Germany) diluted in androgen assay buffer (0.1% BSA and 250 ng/mL cortisol in PBS) was added and mixed briefly. Testosterone antibody (50 µL of 1/200,000; no. R3S07-259, Meridian Life Science Inc., Memphis, TN, USA) diluted in androgen assay buffer was added, and plates were incubated (2 h, 28 °C, with shaking). After washing four times as previously described, 3,3’,5,5’-tetramethylbenzidine (TMB; 120 µL; MilliporeK) was added to each well for 10 min of incubation (dark, room temperature, with shaking). The reaction was stopped with a stop solution (80 µL; 1N sulphuric acid). The absorbance was quantified at 450 nm on a LT-4500 Microplate Absorbance Reader (LabTech, Version 7 2010, Tecan Group Ltd., Switzerland). ELISA readings were analysed using a 4-Parameter Logistic nonlinear regression model on SoftMaxPro Software (Version 7.1, Molecular Devices, CA, USA). Inter- and intra-assay CVs were 16.5 and 4.3%, respectively.

### Measurement of androgens - DHEA assay

ELISA plates (96-well) were coated overnight with 1.84 µg/mL donkey anti-sheep serum IgG (prepared in house from Sheep Serum (Scottish Antibody Production Unit, UK)) at 4°C in 100 mM sodium bicarbonate buffer (100 µL). Plates were washed twice with wash buffer (300 µL), then blocked with blocking buffer (220 µL) for 1 h at room temperature. Standards (between 5 and 320 ng/mL prepared from powdered DHEA (Sigma-Aldrich) in assay buffer), quality controls (prepared in house from powdered DHEA in assay buffer) and samples, diluted ×5 in assay buffer, were added in duplicate to the plate (20 µL) with DHEA-HRP (80 µL of 1/12,500; no. 12-04 Astra Biotech, Germany), diluted in androgen assay buffer, and mixed briefly. DHEA antibody (anti-sheep DHEA-Ab, 50 µL of 1/150,000; gifted from Dr Emad Al-Dujaili, University of Edinburgh) diluted in androgen assay buffer was added and plates were incubated (2 h, 28 °C, with shaking). After washing four times as previously described, TMB (120 µL) was added to each well for 10 min of incubation (dark, room temperature, with shaking). The reaction was stopped with stop solution (80 µL). The absorbance was quantified and ELISA readings were analysed as described above for testosterone. Inter- and intra-assay CVs were 14.7 and 7.6%, respectively.

### Validation of testosterone and DHEA assays

The same set of validations were undertaken on both the androgen assays. The limit of detection (LOD) was determined by running 10 wells of the blank and calculated as two s.d. above the mean value. The limit of quantification (LOQ) was calculated by running the standard curve 12 times, along with two additional lower standards (0.015 and 0.007 ng/mL for T and 2.5 and 1.25 ng/mL for DHEA), plotting the percent coefficient of variation (%CV) of each standard, and determining the concentration point when the %CV was 20% or lower. At a %CV of 20%, this was regarded as the LOQ (Andreasson *et al.* 2015). Thirty-three steroids (estrogens, progestogens, androgens and glucocorticoids) were tested on the assays to determine potential cross-reactivities (listed in Supplementary Data 1, see section on supplementary materials given at the end of this article). Three giant panda urine samples were serially diluted (neat, ×2, ×4, ×8, ×16, ×32, ×64) in assay buffer to determine the parallelism of each assay for the sample type. Six freeze–thaw cycles of both neat urine and pre-diluted urine at ×5 (three urine samples) were analysed to determine the potential effect on the readings. A biological validation was performed with urine samples from the male giant panda SB564. All samples were measured on both the testosterone and DHEA assays, covering two breeding seasons prior to the detection of testicular tumours, the consequent castration, and a 1-year period following castration.

### Statistical analysis

Data were aligned to the day of peak urinary oestrogen concentrations, with the period of interest for this study from 40 days prior to peak until 10 days after peak. This period was then further divided into four phases: anestrus, proestrus, estrus, and post-estrus. Here, anestrus is defined as the period of 40 days before peak until the estrogen concentration reaches two s.d.s above the mean estrogen concentration. Proestrus is the period starting when estrogen becomes two s.d.s above the mean estrogen until 1 day prior to the peak (Kersey *et al.* 2010*a*). Estrus is the day of peak estrogen. Post-estrus is from the day following peak estrogen until 10 days post peak estrogen (Wauters *et al.* 2018).

Statistical analysis was undertaken on GraphPad Prism (Version 7.02, GraphPad Software, Inc.). For ELISA validations, paired two-tailed* t*-tests were undertaken. The hormone data were deemed not normally distributed by a Shapiro–Wilk test, thus statistics were non-parametric. Within the androgens, a Spearman’s correlation compared concentrations obtained by the testosterone and DHEA assays. A Kruskal–Wallis test with Dunn’s Multiple Comparisons compared steroid hormone concentrations across the phases of the period of interest. Significance was defined as *P* < 0.05.

## Results

### Testosterone and DHEA assay assessment

**Figure 1:**
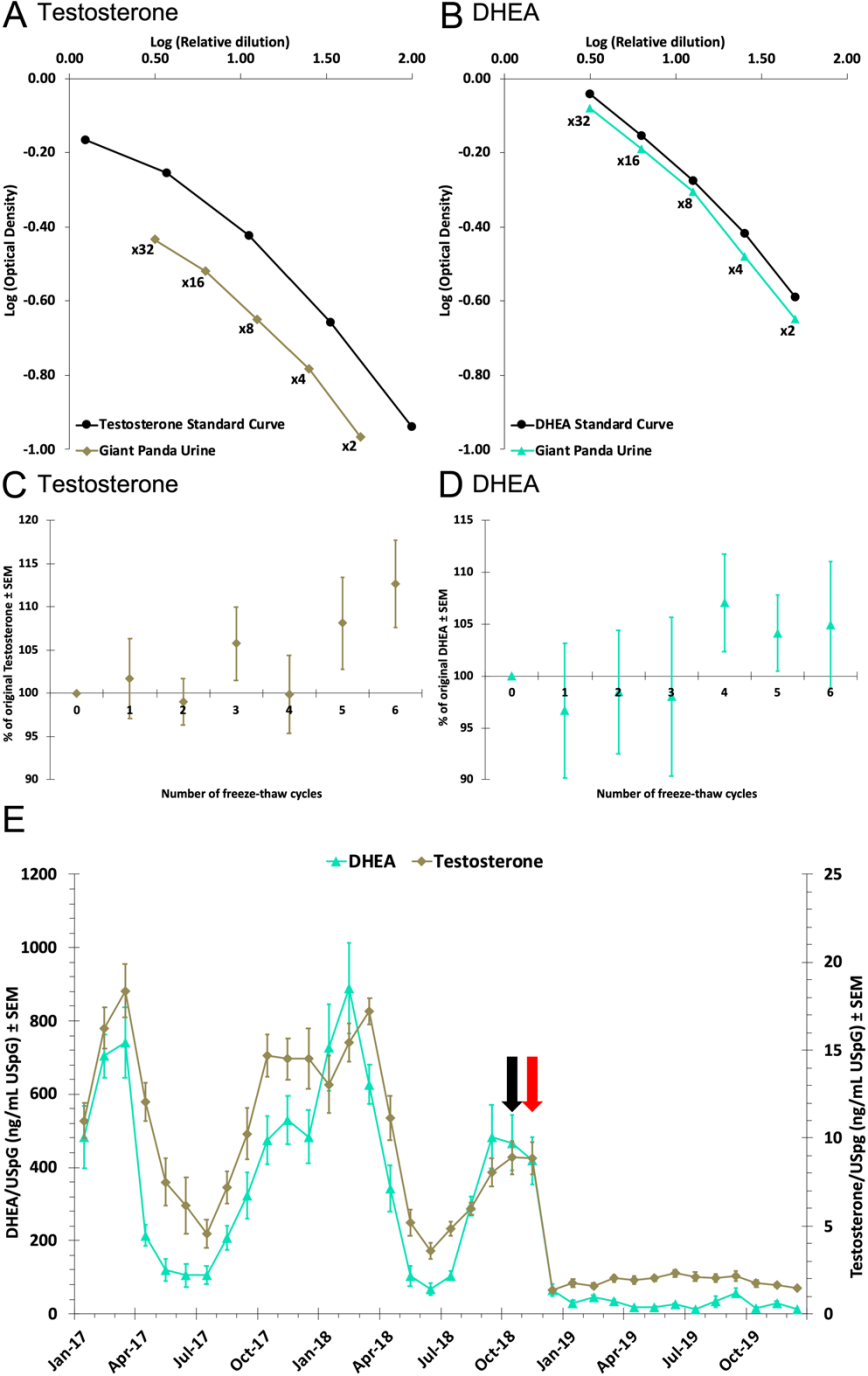
Parallelism assessment of giant panda urine (*n* = 3) in comparison with the testosterone (A) and DHEA (B) standard curves. A good parallelism was achieved for giant panda urine with dilutions between × 2 and × 32. Freeze-thaw cycle assessment of giant panda urine (*n* = 3) for both neat urine and a × 5-pre-diluted urine as tested for both testosterone (C) and DHEA (D) following up to six freeze-thaw cycles. There was no significant difference for either neat or pre-diluted urine at any number of freeze-thaw cycles in comparison to the original hormone concentrations of the samples (testosterone *P* = 0.70; DHEA *P* = 0.91). Monthly average testosterone and DHEA (E) profiles ± s.e.m. for a castrated male giant panda from the 22 months prior to castration until 13 months following castration. The black arrow indicates the month of tumour discovery, the red arrow indicates the month of castration. The November 2018 monthly average is indicative of the intact male, the first urine samples from post-castration were from December 2018.

The LOD of the testosterone assay was calculated as 0.039 ng/mL, and the LOQ as 0.16 ng/mL. The LOD of the DHEA assay was calculated as 0.19 ng/mL, and the LOQ as 0.75 ng/mL. Thirty-three steroid hormones were analysed on both assays to test cross-reactivities. The testosterone assay had the following cross-reactivities against testosterone standards where testosterone is represented as 100%: 69.50% dihydrotestosterone, 19.66% androstenediol, 13.68% 3α-androstanediol, 9.31% dihydroandrosterone, and 6.81% androstenedione; all other steroids were found to have less than 5% cross-reactivity (Supplementary Data 1). The DHEA assay had the following cross-reactivities against DHEA standards where DHEA is represented as 100%: 51.18% DHEA-S, 24.88% androstenedione and 14.44% androsterone; all other steroids were found to have less than 5% cross-reactivity (Supplementary Data 1). Three giant panda urine samples were serially diluted and assessed for their response with both assays. Samples were too dilute at an ×64 dilution; however, good parallelism was observed when comparing the urine to the standard curve at dilutions between ×2 and ×32 (Fig. 1A and B). Three giant panda neat urine and ×5 pre-diluted samples were exposed to six freeze–thaw cycles and analysed with both assays. Neither concentrations obtained with the testosterone assay nor the DHEA assay were affected following six freeze–thaw cycles for either neat urine or pre-diluted urine (Fig. 1C and D; average recovery ± s.e.m. testosterone assay = 104.5 ± 2.4% DHEA assay = 101.5 ± 2.33%); for the testosterone assay, both five and six freeze–thaw cycles demonstrated more deviation from original hormone concentration than lower numbers of freeze–thaw cycles; however, these were not statistically significant.

### Biological validation

The male androgen profiles obtained with the androgen assays indicate seasonality (Fig. 1E). High androgen concentrations were observed on both assays from January to April 2017, and September 2017 to April 2018. Concentrations had started to increase again from August 2018 and remained elevated when testicular tumours were discovered. Following castration, urinary androgen concentrations were significantly decreased (*P* < 0.0001) and remained unchanged from December 2018 through December 2019. This seasonal profile of androgens (testosterone) is fitting with those previously reported for male giant pandas, confirming the biological validity of the assay. The paired seasonal profile obtained with the DHEA assay, and concentration decrease following castration, potentially highlights the major gonadal source of DHEA in male giant pandas.

### Prediction of an impending proestrus

**Figure 2:**
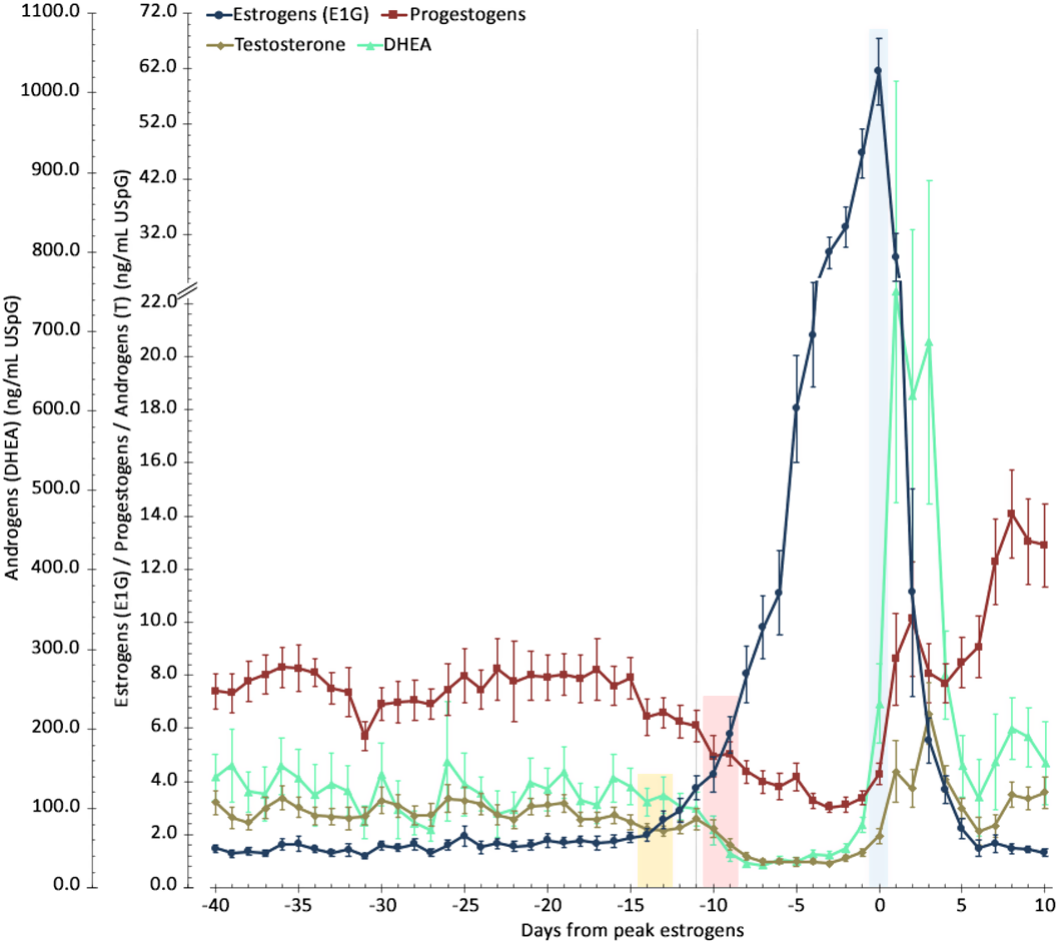
The combined average hormone profiles of estrogens (E), progestogens (P) and androgens (testosterone (T) and DHEA) ± s.e.m. over the period of interest aligned to the day of peak estrogen. Note the split y-axis scale on the E/P/T axis to allow for clear visualization of the combined profiles at the timing of the entry into proestrus, and the separate y-axis for DHEA. The grey line at day -11 indicates the average start of the proestrus period based on when the concentration of estrogen increased 2 s.d. above the mean concentration. The E/P crossover is highlighted in red, occurring between day -10 and -9 following the aforementioned entry into proestrus. The E/T crossover is highlighted in yellow, occurring between day -14 and -13. The day of peak estrus (day 0) is highlighted in blue.

The average profile ± s.e.m. of estrogens (E), progestogens (P), and androgens (T and DHEA, respectively representing the results obtained with the testosterone and DHEA assays) is shown in Fig. 2 aligned to the day of peak estrogens. Table 3 highlights an increase in estrogen concentrations from anestrus, into proestrus and then estrus – with estrus concentrations 3.5 -times greater than proestrus. Conversely, progestogens and androgens showed significant decreases, by approximately two-fold, between anestrus and proestrus (*P* < 0.0001 for all). The two androgens were correlated (r = 0.64), with DHEA concentrations on average being 50 times higher than testosterone concentrations.

**Table 3:** Summary of the average hormone concentrations (ng/mL USpG) for each hormone (E, P, T, and DHEA with the s.e.m.) during each phase within the period of interest.

	Anestrus	Proestrus	Estrus	Post-estrus
E/USpG	1.68 (0.05)^a,b,c^	17.47 (1.17)^a,d^	61.51 (6.07)^b,e^	5.85 (0.87)^c,d,e^
P/USpG	7.50 (0.15)^a,b,c^	4.06 (0.14)^a,d^	4.29 (0.39)^b,e^	10.56 (0.50)^c,d,e^
T/USpG	2.82 (0.07)^a,b^	1.33 (0.07)^a,c^	1.95 (0.31)^b,d^	3.64 (0.23)^c,d^
DHEA/USpG	120.69 (5.93)^a,b^	49.62 (3.15)^a,c,d^	231.65 (49.71)^c^	322.77 (43.19)^b,d^

The superscripts indicate significant differences between phases for each hormone.

Proestrus begins at day −11 from peak (range: −8 to −14 days) when estrogen is increased two s.d.s above the mean concentration, while there is a decreasing trend for progestogens and the androgens. When assessing the combined profile, there are two crossover points that can be identified. Progestogen concentrations begin to decrease from −15 days before peak estrogen, with the E/P crossover (where the ratio between the two hormones reaches 1.0) occurring at −9 days from peak estrogen (range: −7 to −11 days). Alternatively considering testosterone, the E/T crossover occurs at −13 days from peak estrogen (range: −9 to −17). Testosterone has a 4-day advantage over progestogen at determining impending proestrus (Fig. 2).

### Prediction of peak fertility

At estrus, all hormones increased from their proestrus concentrations. Aside from estrogen, this increase was also significant for DHEA (*P* < 0.0001). There was a clear trend towards an increase in both testosterone and progestogen. In individual cycles, the increase could be more clearly observed when there were a greater number of samples collected on the day of estrus.

Using the hormone profiles to predict peak estrus, the distinct significant estrus increase in DHEA (with concentrations 4.7 times greater at estrus than during proestrus) provides a marked hormonal change associated with estrus and confirmation of peak estrogen.

There is some cycle-to-cycle variation of DHEA concentrations, including within individual females – thus the magnitude of the increase at estrus should be considered in relation to the proestrus DHEA concentrations in each individual cycle. Proestrus concentrations ranged between 16.30 and 93.95 ng/mL USpG, and the concentration at estrus ranged between 21.14 and 759.54 ng/mL USpG (Supplementary Data 2). Following estrus, DHEA showed peak concentrations between +1 and +3 days from peak estrogen and remained elevated for up to +4 days from peak estrogen before decreasing.

## Discussion

This study identifies the advantage of combining the monitoring of estrogens (E), progestogens (P), and androgens (testosterone and DHEA) in the female giant panda for the early prediction of both impending estrus and the confirmation of peak estrogen, indicating the start of the fertile window. Androgens have not previously been measured in female giant pandas. This novel finding has great potential for use across captive giant panda breeding programmes enhancing how estrus is monitored by advancing early warning of proestrus and confirming estrus. This is particularly important because female giant pandas have one chance of fertility per year.

We developed and validated testosterone and DHEA assays to measure androgens in giant panda urine. Testosterone metabolites have been previously measured in male giant panda urine (MacDonald *et al.* 2006, Steinman *et al.* 2006, Kersey *et al.* 2010*c*, Gocinski *et al.* 2018); however, the biological validation step in our study provides the first measurement of male giant panda urinary androgens with a DHEA assay. In the intact male, DHEA showed a seasonal profile close to that of testosterone. Following castration, both androgens significantly decreased and showed no annual seasonal profile. This highlights that the gonads are the major source of DHEA in the giant panda, in-keeping with species such as rats (Belanger *et al.* 1989, Belanger *et al.* 1990, van Weeren *et al.* 1992), guinea pigs (Rivarola *et al.* 1968, Belanger *et al.* 1989), rabbits (Cutler *et al.* 1978, Schiebinger *et al.* 1981), dogs (Cutler *et al.* 1978, Schiebinger *et al.* 1981, Mongillo *et al.* 2014) and cattle (Marinelli *et al.* 2007). This is in contrast to species such as humans (Labrie *et al.* 2005), primates (western lowland gorillas (Edes 2017)), chimpanzees (Seraphin *et al.* 2008, Blevins *et al.* 2013), bonobos (Behringer *et al.* 2012), macaques (Muehlenbein *et al.* 2002), red squirrels (Boonstra *et al.* 2008), American martens (Boonstra *et al.* 2018) and both Gray and Harbor seals (Gundlach *et al.* 2018) which have a primarily adrenal source for DHEA. Other *Ursid* species have not had the source of their DHEA investigated. In the species with mainly adrenal DHEA production, DHEA concentrations and profiles are often studied in relation to stress or injury response. As giant pandas show mainly gonadal production, we believe the observed profiles are not likely related to potential stressors or injury.

In determining a predictive biomarker for proestrus, the E/T crossover occurred an average of 4 days prior to the E/P crossover. Current methods of monitoring the breeding season focus on measuring estrogen and progestogen to indicate proestrus and future fertility. The addition of androgen (e.g. testosterone) measurements could extend this indicative window by 4 days to allow additional time for preparation. In steroidogenesis, androgens are converted to estrogens via aromatase. With the significant increase in estrogen concentrations between the anestrus and proestrus periods, it is perhaps logical to expect a decrease in the concentrations of precursors of estrogens. With the rapid increase in estrogen, androgens consistently remain low for the duration of proestrus.

Follicles have differing steroid-producing capabilities as they develop (Rodgers 1990, Mason & Franks 1997, Chryssikopoulos 2000, Wood & Strauss 2002). Detailed studies on giant panda ovaries or follicles have not been undertaken, but in the Hokkaido brown bear, steroidogenic enzymes have been localised within follicles. Aromatase was not detected in follicles less than 6 mm in diameter. Follicles greater than 6 mm in diameter expressed 17α-hydroxylase in the theca interna and aromatase in the granulosa cells (Araki *et al.* 1996). Hokkaido brown bears have one major follicular wave per breeding season, where one or two follicles grow larger than 6 mm in diameter, at which point they have gained the ability for estrogen synthesis (Torii *et al.* 2020). Torii *et al.* (2020) suggest that Hokkaido brown bear follicles take around 2 weeks to grow to a preovulatory size during the major follicular wave. This may also be the case in the giant panda and at the observed E/T crossover the follicles have now likely developed to the required stage and size for aromatase expression. This crossover occurs at an average of 13 days before peak estrogen, fitting with those observations of 2 weeks of growth to preovulatory size in the Hokkaido brown bear (Torii *et al.* 2020). In agreement with these observations, ultrasound imaging of follicular development in the giant panda shows 6 mm follicles at the start of proestrus (Hildebrandt *et al.* 2006).

During proestrus, there are no previous reports of androgen concentrations in giant pandas. In the polar bear, fecal estradiol concentrations were unchanged across periods where breeding behaviours were shown by females, but both fecal and urinary testosterone showed changes associated with breeding behaviours (Stoops *et al.* 2012, Knott *et al.* 2017). In the sun bear,Schwarzenberger *et al.* (2004) found that fecal epiandrosterone was increased only over the follicular phase and decreased prior to the luteal pregnanediol-glucuronide increase. Considering non-*ursid* species, few publications have considered the measurement of androgens in females. In the dog, fecal testosterone mirrored estradiol concentrations (Gudermuth *et al.* 1998, Concannon 2009). Studies in mares, while not stating proestrus androgen concentrations, have shown testosterone or androstenedione concentrations to increase coincident with ovulation (Silberzahn *et al.* 1978, Meinecke *et al.* 1987). In mares,Meinecke *et al.* (1987) showed an increasing plasma DHEA concentration from 7 days prior to ovulation that peaked the day before ovulation, followed by a steady 10-day decrease that paralleled estrone and estradiol.Meinecke *et al.* (1987) suggested the DHEA increase was from an extra-follicular source, for example from the adrenal gland. Conversely, we did not observe parallel increases in androgens and estrogen during proestrus, with decreased androgen concentrations whilst estrogen rapidly increased. From our DHEA ELISA validation, we know that in the giant panda, DHEA is primarily produced in the gonads, thus it may be the case that this difference in profiles relates to the primary source of the hormone. Additionally, as we observed a similar pattern between the progestogen and androgen profiles it is possible that these hormones are not differentially controlled. There is however a gap in the literature regarding androgen assessment in estrus monitoring.

At peak estrus in the giant panda, DHEA has the most potential in advancing the interpretation of the hormone profiles, as it increases significantly on the day of peak estrogen. Concentrations obtained with the DHEA assay remained elevated until 5 days post-estrus. From the day of peak estrogen to +4 days from the peak, these DHEA concentrations were on average 3.4 times higher than anestrus concentrations. Whilst both testosterone and progestogen tend to be elevated during this time frame, neither is elevated to the same extent as DHEA.

We speculate that DHEA could be used as an alternative measure to LH for the detection of peak fertility. While LH has been assayed in giant panda urine, the results are variable and the method is not widely available. LH was shown to peak between 3 h 37 min and 61 h 51 min following peak estrogen (Cai *et al.* 2017). The wide variation between individuals may have been related to sampling frequency. In the hypothalamic–pituitary–gonadal axis, pituitary LH acts at the ovarian level to produce androgens, which act as precursors for estrogens (Chimote &Chimote 2018). Generally, DHEA is known to be much more abundant in circulation than other androgens, including testosterone, and this may explain the more noticeable change in DHEA hormone concentrations (Labrie *et al.* 2005). The increase of DHEA on the day of peak estrogen may be a downstream result of the start of the LH surge. LH measurements would need to be made in parallel to DHEA to be able to further investigate this suggestion.

In conclusion, additional monitoring of androgens across the breeding season in the giant panda not only provides an increased advanced warning of an impending estrus (through the measurement of testosterone) but also gives advanced indications of peak estrogen concentration (through DHEA). Together, these can aid in preparations for breeding. AIs are dependent on endocrinological monitoring and are known to have a lower success rate than NM (18.5% compared with 60.7% birth rate (Li *et al.* 2017)). With the advancement in predicting estrus that androgens provide, there may be the potential to increase this success rate. This may allow pandas who may not have success with NM to increase the genetic variability of the captive breeding gene pool through AI. Being a monoestrus seasonally breeding species, gaining advanced warning to prepare for breeding and confirmation of optimal fertility is of great importance. Optimisation of estrus monitoring taking into consideration these findings could provide benefit to giant panda breeding worldwide.

## Supplementary Material

Supplementary Data 1: The thirty-three steroids tested on both the testosterone and DHEA assays and their cross-reactivity percentages from the assays.

Supplementary Data 2: The individual DHEA and E1G paired profiles highlighting the individual variation that the hormones can show between cycles and pandas, and grouped into cycle outcome. Note the differences in the y-axes of each profile. A green arrow indicates the timing of mating or artificial insemination (AI) which resulted in a successful pregnancy. An orange arrow indicates the timing of a mating or AI which did not result in a successful pregnancy (non-birth). No arrow indicates a pseudopregnant cycle (no mating or AI). Two arrows indicates the performance of two mating or AI procedures

## Declaration of interest

The authors declare no conflicts of interest. W Colin Duncan is an Associate Editor of *Reproduction and Fertility.* W Colin Duncan was not involved in the review or editorial process for this paper, on which he is listed as an author.

## Funding

This work did not receive any specific grant from any funding agency in the public, commercial, or not-for-profit sector.

## Author contribution statement

All authors undertook experimental design and conceptualisation, and were involved with writing and editing of the manuscript. K S W, J W, I V and S G were involved with data curation. K S W, J W, W C D, I V and A M undertook data analysis.
