# Supplementary material for: The novel use of urinary androgens to optimise detection of the fertile window in giant pandas
Source: Reprod Fertil. 2022 Jun 30;3(3):122–32. doi: 10.1530/RAF-22-0031 (PMC9354564; doi:10.1530/RAF-22-0031)
Supplement: Supplementary Data 1: The thirty-three steroids tested on both the testosterone and DHEA assays and their cross-reactivity percentages from the assays. [file supplementary_table_1.pdf]

| Steroid | | % Cross Reactivity | |
|---|---|---|---|
| | | Testosterone | DHEA |
| ***Androgens*** | 11-Ketotestosterone | 2.48 | 0.22 |
| | 3α-androstanediol | 13.68 | 0.62 |
| | 3β-androstanediol | 4.62 | 1.25 |
| | 5α-androsten-3β-17β-diol | 19.66 | 0.67 |
| | Androstenedione | 6.81 | 24.88 |
| | Androsterone | 0.34 | 14.44 |
| | DHEA | 0.45 | 100.00 |
| | DHEA-S | 0.21 | 51.18 |
| | Dihydroandrosterone | 9.31 | 0.74 |
| | Dihydrotestosterone | 69.50 | 0.97 |
| | Etiocholanolone | 0.20 | 1.73 |
| | Testosterone | 100.00 | 0.54 |
| ***Estrogens*** | Estradiol | 0.85 | 0.50 |
| | Estriol | 0.20 | 0.29 |
| | Estrone | 0.33 | 1.60 |
| | Estrone-3-Glucuronide | 0.21 | 0.16 |
| ***Glucocorticoids*** | 11-deoxycortisol | 0.28 | 0.48 |
| | Corticosterone | 0.19 | 0.16 |
| | Cortisol | 0.19 | 0.17 |
| | Cortisone | 0.17 | 0.18 |
| | Deoxycorticosterone | 0.21 | 0.31 |
| ***Progestagens*** | 17α-hydroxypregnanolone | 0.81 | 0.00 |
| | 17α-hydroxypregnenolone | 0.17 | 0.22 |
| | 17α-hydroxyprogesterone | 0.19 | 0.16 |
| | 21-Hydroxyprogesterone | 0.37 | 0.20 |
| | 5α-pregnane-3α-ol-20-one | 0.17 | 0.00 |
| | 5α-pregnane-3β-1,20dione | 0.00 | 1.23 |
| | 5α-pregnane-3,6-ol-20-dior | 0.00 | 1.46 |
| | 5β-pregnane-3-20-dione | 0.34 | 0.28 |
| | Pregnanediol | 0.33 | 0.00 |
| | Pregnanolone | 0.19 | 0.23 |
| | Pregnenolone | 0.28 | 0.56 |
| | Progesterone | 0.49 | 0.82 |
