# Supplementary figures and images for: The novel use of urinary androgens to optimise detection of the fertile window in giant pandas
Source: Reprod Fertil. 2022 Jun 30;3(3):122–32. doi: 10.1530/RAF-22-0031 (PMC9354564; doi:10.1530/RAF-22-0031)

● Estrogens ▲ Androgens (DHEA)

PREGNANCY

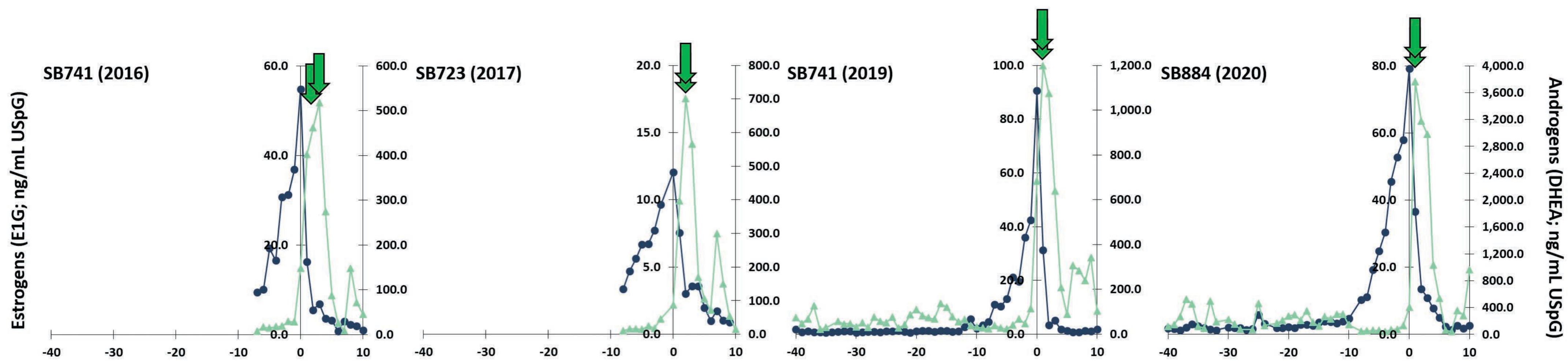

PSEUDOPREGNANCY

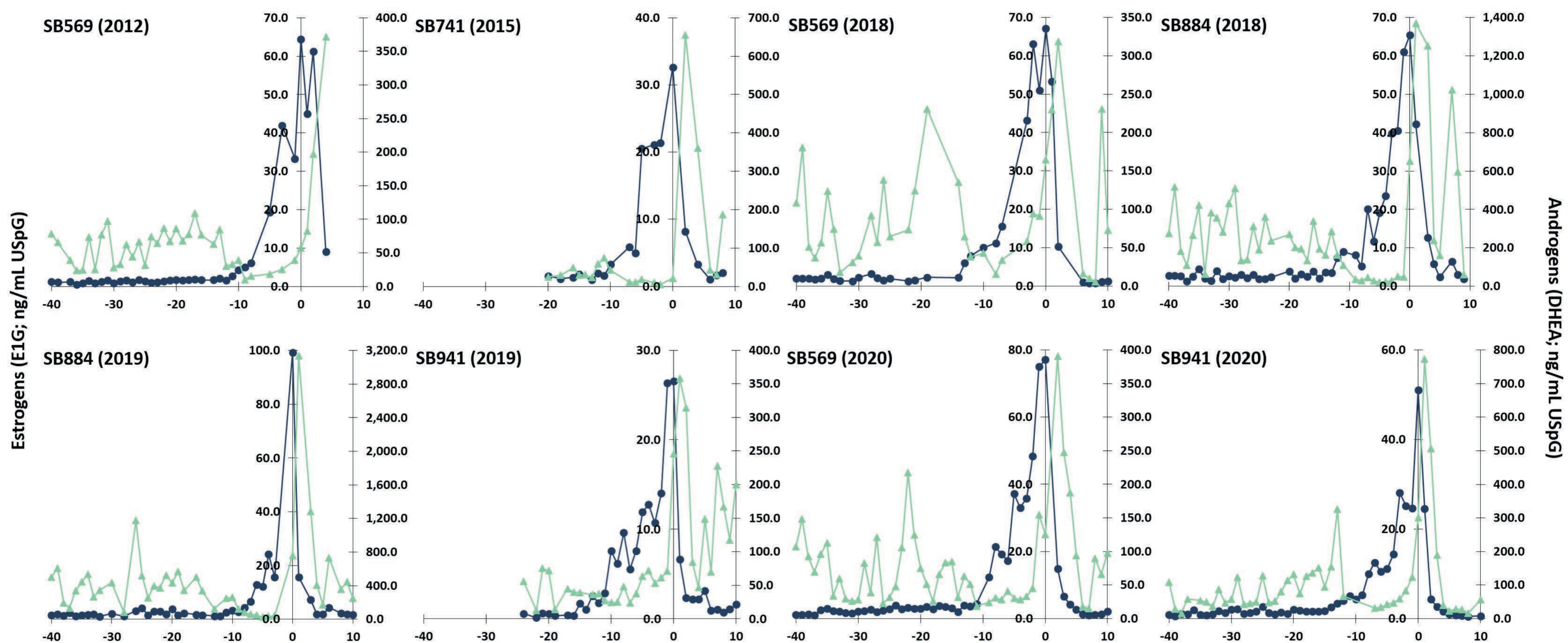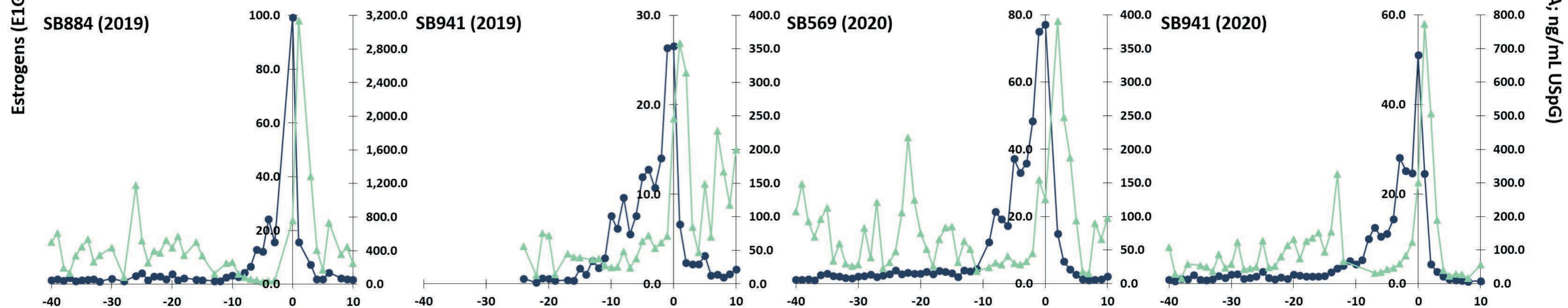

NON BIRTH

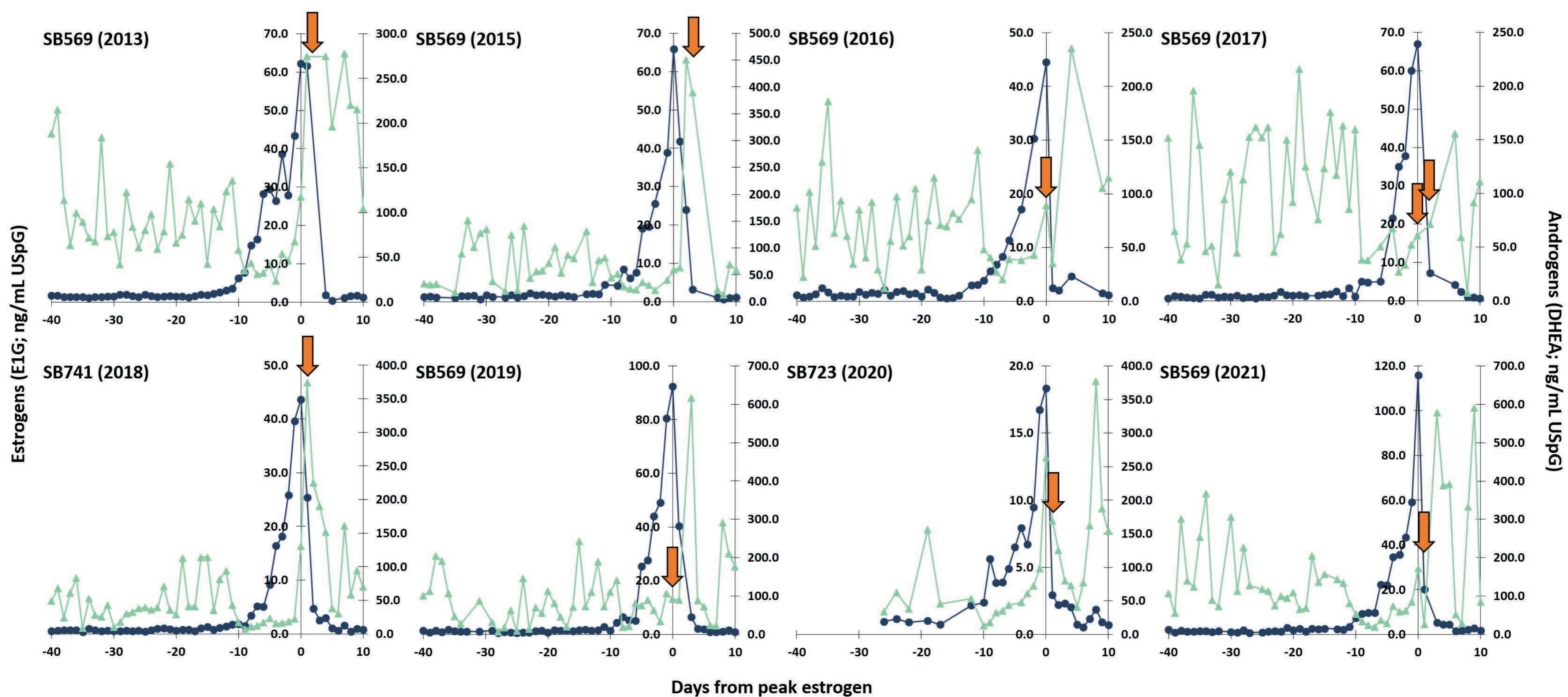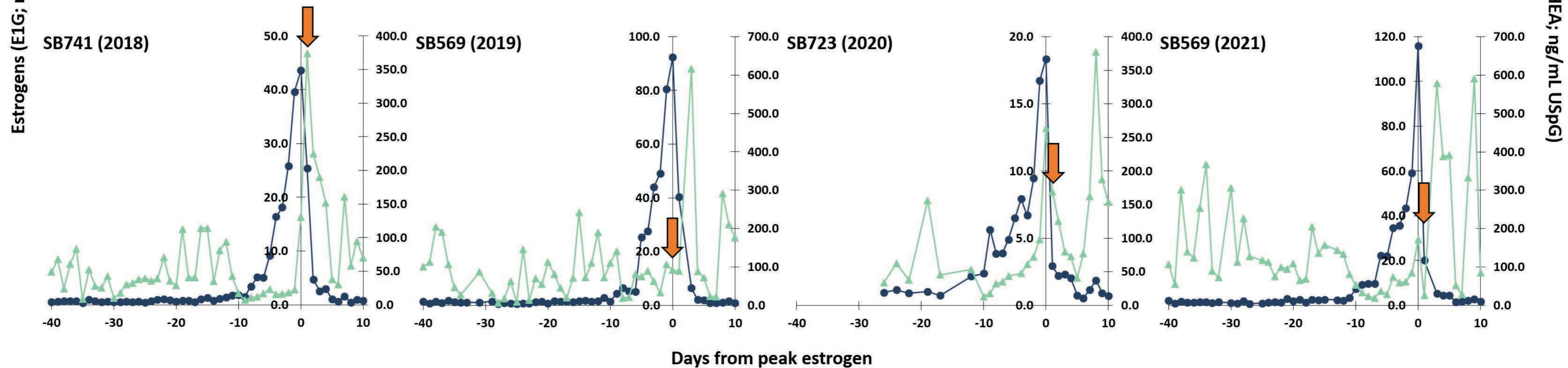

Days from peak estrogen

Supplement: Supplementary Data 2: The individual DHEA and E1G paired profiles highlighting the individual variation that the hormones can show between cycles and pandas, and grouped into cycle outcome. Note the differences in the y-axes of each profile. A green arrow indicates the timing of mating or artificial [file supplementary_figure_1.pdf]
